# Emergency department extracorporeal membrane oxygenation may also include noncardiac arrest patients

**DOI:** 10.3906/sag-2004-308

**Published:** 2021-04-30

**Authors:** Yunus Emre ÖZLÜER, Mücahit AVCİL, Duygu EGE, Kezban ŞEKER YAŞAR

**Affiliations:** 1 Department of Emergency Medicine, Faculty of Medicine, Adnan Menderes University, Aydın Turkey

**Keywords:** Extracorporeal membrane oxygenation, emergency department, cardiac arrest, resuscitation, critical care

## Abstract

**Background/aim:**

The primary purpose of this study is to report the experience on the extracorporeal membrane oxygenation (ECMO) process for patients in the critical care unit (CCU) of an emergency department of a tertiary hospital in Turkey, from cannulation to decannulation, including follow-up procedures.

**Materials and methods:**

This retrospective and observational study included eight patients who received ECMO from January 2018 to January 2020. We evaluated the demographics, indications for ECMO, laboratory values, Respiratory ECMO Survival Prediction, Survival After Veno-Arterial ECMO and ECMO net scores, the management process, and patient outcomes. Blood gas analyses done after the first hour of ECMO initiation and the reevaluation of the patients’ Acute Physiology and Chronic Health Evaluation (APACHE) II and Sequential Organ Failure Assessment (SOFA) scores in the 24th hour of ECMO were recorded.

**Results:**

The mean age was 52.7 ± 14.2 years. The median duration of the ECMO run was 81 (min–max: 4–267) h, and the mean length of CCU stay was 10.2 ± 6.7 days. Of the 8 patients studied, 5 (62.5%) had veno-arterial and 3 (37.5%) had veno-venous ECMO. Three patients were successfully weaned (37.5%). The overall survival-to-discharge rate was 25%. Carbon dioxide levels were significantly decreased 1 h after ECMO initiation (P = 0.038) as well as the need for vasopressors. Lactate levels were lower in decannulated patients. Changes in the APACHE II score were more consistent with the clinical deterioration in patients than SOFA score changes were.

**Conclusions:**

In the early phase of ECMO, vital signs improve, and the need for vasopressors and carbon dioxide levels decrease. Thus, CCUs in Emergency Departments with ECMO capabilities could potentially be designed, and emergency department ECMO algorithms could be tailored for critically ill patients in addition to out-of-hospital cardiac arrest patients.

## 1. Introduction

Extracorporeal membrane oxygenation (ECMO) is a temporary method for restoring blood flow and extracorporeal gas exchange while the heart and/or the lungs rest until the decision for definitive treatment is made [1]. Since Bartlett et al. first used ECMO successfully in a newborn with respiratory failure in 1975, it has been a widespread utility in acute cardiac failure and respiratory failure due to Acute Respiratory Distress Syndrome (ARDS) [2–4]. Thus, ECMO has become a vital part of the clinical practices of cardiovascular surgery and anesthesiology. As per recent cardiopulmonary resuscitation (CPR) guidelines [5], extracorporeal (E-CPR) may be considered an alternative to conventional CPR, and extracorporeal life support has gained a new perspective. Furthermore, emergency physician-initiated ECMO programs for out-of-hospital cardiac arrest (OHCA) patients have been built in this decade [6]. However, these programs do not include patients other than OHCA. 

Our study aims to investigate whether the spectrum of these programs should include other critically ill patients by reporting the experience of using ECMO in our emergency department (ED).

## 2. Materials and methods

### 2.1. Study population

This study retrospectively analyzed eight ECMO cases from January 2018 to January 2020 to evaluate demographics, indications for ECMO, the management process, and the patients’ outcomes. The study was approved by the Aydın Adnan Menderes University Committee of Ethics (Approval No. 2019/109). The ED of Adnan Menderes University Hospital is a tertiary center with annual admittance of 100,000 patients. Our ED is unique in that it has an intensive care unit (ICU) that is run by emergency physicians and has a six-bed capacity. It has been serving as a CCU since 2012. Our ED cares for high-risk referrals from all nearby cities.

### 2.2. Patient selection

The patients were selected according to the Extracorporeal Life Support Organization (ELSO) guidelines [7]. Cardiogenic shock caused by myocardial infarction, sepsis, or intoxication that did not resolve with the conventional therapies comprised the main indications for veno-arterial (VA) ECMO (Table 1). As for selecting patients for veno-venous (VV) ECMO, patients with severe hypoxic respiratory failure, refractory to the maximal medical, and ventilatory support were determined eligible for this therapy. In both VA and VV ECMO patients, we strongly considered whether the patient had a potentially reversible condition. 

**Table 1:** The demographic and clinical data of the patients.

	Case 1	Case 2	Case 3	Case 4	Case 5	Case 6	Case 7	Case 8	Mean ± SD	Median
Age	61	60	74	30	53	41	61	42	52.7 ± 14.2	56.5
Sex	M	M	M	M	M	M	F	M	NA	NA
Indication for ECMO	Intoxication	ARDS	Intoxication	ARDS	STEMI	Intoxication	ARDS	ARDS	NA	NA
ECMO type	VA	VVSwitched to VAV	VA	VA	VA	VV	VV	VASwitched to VV	NA	NA
ECMO duration (h)	9.5	20	4.2	72.7	90	186.1	267.5	252.8	112.8 ± 108.2	81.3
CCU stay (d)	3	4	1	13	18	14	18	11	10.2 ± 6.7	12.0
Weaning from ECMO	No	No	No	No	Yes	Yes	Yes	No	NA	NA
Outcome	Exitus	Exitus	Exitus	Exitus	Discharged	Discharged	Exitus	Exitus	NA	NA
Complication	Minorbleeding	-	-	-	GI bleeding	-	HIT	-	NA	NA
RESP	-	0	-	-	-	5	4	-	3.0 ± 2.6	4.0
SAVE	–8	-	–12	3	–2	-	-	–4	–4.6 ± 5.7	–4.0
ECMOnet	-	4.5	-	-	-	2	4	-	3.5 ± 1.3	4.0
Pre-SOFA	6	11	3	9	13	8	13	12	9.4 ± 3.6	10.0
Post-SOFA	11	15	11	13	16	8	15	8	12.1 ± 3.1	12.0
Pre-APACHE II	24	33	22	24	22	18	23	20	23.2 ± 4.4	22.5
Post-APACHE II	30	33	46	30	20	15	24	17	26.9 ± 10.1	27.0

ARDS: acute respiratory distress syndrome, STEMI: ST-elevation myocardial infarction, VV: veno-venous, VA: veno-arterial, VAV: veno-arterio-venous, GI: gastrointestinal, HIT: heparin-induced thrombocytopenia, RESP: respiratory ECMO survival prediction, SAVE: survival after veno-arterial ECMO, Pre-SOFA: Sequential Organ Failure Assessment Score before ECMO, Post-SOFA: Sequential Organ Failure Assessment Score after 24 h of ECMO, Pre-APACHE II: Acute Physiology and Chronic Health Evaluation II Score before ECMO, Post-APACHE II: Acute Physiology and Chronic Health Evaluation II Score after 24 h of ECMO, NA: not Applicable.

Patients with severe irreversible organ failures, incurable end-stage metastatic malignancies or severe coagulopathy, and patients older than 75 were considered not suitable candidates for ECMO.

### 2.3. ECMO procedure and care 

All cannulations were performed by emergency physicians, either with Seldinger’s method under ultrasonographic guidance or femoral cut-down. VV ECMO cannulations were made through the right internal jugular vein and the femoral vein. Cannulations for VA ECMO were performed through the femoral vein and the common femoral artery. A 7-Fr sheath as a back-flow cannula was placed in the superficial femoral artery in all VA ECMO patients. For the most part, 19–21-Fr cannulas were used for arterial access, and 21–23-Fr cannulas were used for venous drainage. Activated clotting time monitoring was performed hourly until it reached a steady-state and every 6 h after settling. Moreover, hemoglobin, hematocrit, platelet, and fibrinogen levels were monitored daily.

In all the cases, we used centrifugal pumps (Maquet Rotaflow, Maquet Cardiopulmonary AG, Hirrlingen, Germany) with hollow-fiber membrane oxygenators (Maquet Quadrox-iD, Maquet Cardiopulmonary AG, Hirrligen, Germany). The initial pump flow rate (FR) was set to provide approximately two-thirds of the patient’s native cardiac output (CO) in the VV ECMO, and the sweep gas rate was set to two times the pump FR. In cases of VA ECMO, we initially aimed to set a blood FR of 2–3 L/min to provide partial assistance and 4–6 L/min when there was no native CO, such as in asystole. CO was measured, calculated, and recorded daily by echocardiography in all the patients.

All of the patients enrolled in this study were mechanically ventilated. Mechanical ventilatory settings were adjusted according to either the “lung-protective ventilation” strategy (Vt 4–6 mL/kg, PEEP 8–10 cm H₂O, PIP 25–30 cm H₂O, Rate 15–20/min) or airway pressure release ventilation mode (inspiration time 4.5 s, expiration time 0.5 s, high pressure up to 30 cm H₂O, low pressure 0 cm H₂O) in patients receiving VV ECMO. Conventional mechanical ventilatory settings (e.g., pressure SIMV mode with tidal volume 6–8 mL/kg, PEEP 5–8 cm H₂O, PIP 15–20 cm H₂O, rate 12–14/min) were mostly used in VA ECMO patients.

### 2.4. Weaning from ECMO

When weaning VV ECMO patients, we firstly ensured that lung-protective ventilation was no longer needed. We then turned off the gas sweep, suspended the oxygen flow into the oxygenator, and monitored the patient closely to detect any increase in respiratory effort. After 6 h of stability, the patient was considered ready for decannulation. The weaning trial for VA ECMO patients was done under the guidance of transthoracic echocardiography. The baseline left ventricle functions were assessed before the weaning trial, followed by the pump rate being decreased 1 L/min. Reassessment of the left ventricular functioning was made, and an improvement in CO was sought. As long as the left ventricle allowed, and if the CO was satisfactory, the decrements of 1 L/min continued until an FR of 1.5 L/min was achieved. The cannulas were locked with heparin and clamped for 12 h, mostly overnight. Left ventricle functions were reassessed, and when there were no impairments and the patient was stabilized, we proceeded to the decannulation phase. 

Venous cannulas were simply withdrawn, and the pressure was applied to the site for 20 min. However, surgical methods, such as vascular wall repair and femoral fascia suture, were used in the removal of the arterial cannulas. We also used a Proglide vascular closure device (Abbott Vascular, Redwood City, CA, USA) in one of our VA ECMO cases. Postdecannulation venous and arterial Doppler studies were performed daily at the bedside.

### 2.5. Data collection

After the decision for the initiation of ECMO was made, Respiratory ECMO Survival Prediction (RESP) and ECMO net scores were calculated in the patients who had VV ECMO; and Survival After Veno-Arterial ECMO (SAVE) scores were calculated in patients who had VA ECMO to predict the rate of in-hospital survival. Blood gas analyses including lactate levels from before and after the first hour of ECMO initiation, and the reevaluation of the patients’ APACHE II and SOFA scores at the 24th hour of ECMO were recorded.

### 2.6. Data analysis

Statistical analysis was performed using SPSS 20 for Windows (SPSS Inc., Chicago, IL, USA). The demographic and clinical data, such as laboratory values, vasoactive drug infusion rates, and clinical scoring systems, were described with mean ± SD and percentages, and ECMO run duration was described with median (min–max). The Wilcoxon signed-rank and paired samples t-test were used to describe the effect of ECMO on laboratory values and clinical scorings. 

## 3. Results

In our study, a total of 8 patients, 7 males and 1 female, were included. The mean age was 52.7 ± 14.2 years. The median duration of the ECMO run was 81 (min–max: 4–267) h, and the mean length of stay in the CCU was 10.2 ± 6.7 days. The demographic and detailed clinical data are presented in Table 1. 

VA ECMO was performed in 5 cases (62.5%), and 3 patients were supported with VV ECMO (37.5%). In addition, a switch to veno-arterio-venous (VAV) ECMO from VV ECMO was performed in one patient who developed cardiogenic shock while suffering from ARDS; and one patient was changed from VA ECMO to VV ECMO due to the resolution of cardiogenic shock associated with ARDS (Table 1). These patients’ ECMO type groups were specified according to the type applied first. 

The primary indication for ECMO was ARDS in 4 (50%) patients, intoxication in 2 (25%) patients, sepsis in 1 (12.5%) patient, and ST-Elevation myocardial infarction (STEMI) in 1 (12.5%) patient (Table 1). Among patients with ARDS, one patient had fat overload syndrome as a complication of intravenous lipid emulsion therapy for suicidal benzodiazepine ingestion; one patient had H1N1 pneumonia; one patient had invasive pulmonary aspergillosis, and one patient had bacterial pneumonia caused by *P. aeruginosa.* Among the intoxicated patients, one patient’s intoxication was caused by methanol abuse, and the other had a digoxin overdose.

The SAVE scores of the VA ECMO and the RESP and ECMO net scores of the VV ECMO patients are also presented in Table 1. Three patients (37.5%) were weaned from ECMO. One of the weaned patients developed heparin-induced thrombocytopenia after decannulation and subsequently died. The remaining 2 patients were discharged (25%) neurologically intact. The overall survival to the decannulation rate was 37.5%, and the survival-to-discharge rate was 25%.

Mean pH increased from 7.20 ± 0.17 to 7.30 ± 0.15, mean pO2 levels increased from 90.4 ± 40.6 mmHg to 127.8 ± 34.9 mmHg, and mean pCO2 levels decreased from 62.1 ± 30.2 mmHg to 39.1 ± 12.8 mmHg within the first hour in ECMO support. This decrease in pCO2 levels was statistically significant (P = 0.038). Lactate levels after the first hour of ECMO increased in four of eight patients. A decrease in lactate levels after the first hour of ECMO was recorded in three patients who were able to be decannulated (Table 2).

**Table 2:** Initial and 1 h after the ECMO initiation blood gas analyses of the patients.

	Case 1	Case 2	Case 3	Case 4	Case 5	Case 6	Case 7	Case 8	Mean ± SD	Median
Pre-pH	6.93	6.96	7.25	7.13	7.37	7.34	7.31	7.34	7.20 ± 0.17	7.28
Post-pH	7.09	7.26	7.17	7.22	7.38	7.55	7.29	7.49	7.30 ± 0.15	7.27
Pre-O₂ (mmHg)	47.3	69.6	70.0	180.0	99.0	68.9	83.3	105.0	90.4 ± 40.6	76.7
Post-O₂ (mmHg)	57.0	82.7	364.0	133.0	84.9	97.8	93.1	110.0	127.8 ± 97.9	95.4
Pre-CO₂ (mmHg)	108.1	101.0	49.1	75.0	30.7	48.7	26.5	57.8	62.1 ± 30.2	53.4
Post-CO₂ (mmHg)	62.0	31.2	50.9	46.0	25.0	27.9	32.5	37.4	39.1 ± 12.8	34.9
Prelac (mmol/L)	12.8	4.3	1.7	1.0	1.6	1.8	4.9	3,0	3.9 ± 3.8	2.4
Postlac (mmol/L)	17.0	19	10.7	2.7	1.4	1.6	4.7	2.1	7.4 ± 7.2	3.7

Seven patients needed vasopressor/positive inotropic agent support. Mean noradrenaline infusion rates were 36.76 ± 30.23 and 34.55 ± 27.12 µg/min, mean adrenaline infusion rates were 0.10±0.10 and 0.97±0.76 µg/kg/min, and mean dopamine infusion rates were 27.60 ± 33.48 and 5.12 ± 7.24 µg/kg/min before and after 1 h of ECMO initiation, respectively (Table 3).

**Table 3:** Doses of vasotropic agents and mean main arterial pressure of the patients before and 1 h after ECMO.

	Case 1	Case 2	Case 3	Case 4	Case 5	Case 6	Case 7	Case 8
Pre-MAP (mmHg)	50	66.7	74	76.7	53.3	103.3	100	90
Post-MAP (mmHg)	76.7	86.7	66	68.3	70	90	90	93.3
Prenoradrenaline	63.9	53.2	0	6.6	66.6	-	66.6	37.2
Postnoradrenaline	63.9	53.2	36.9	13.2	66.6	-	42.6	0
Preadrenaline	0.25	0.10	-	-	0.07	-	0.01	-
Postadrenaline	0.20	0.11	0.05	-	0.03	-	-	-
Predopamine	51.3	-	-	-	-	-	-	3.9
Postdopamine	10.2	-	-	-	-	-	-	0

Noradrenaline: µg/min, adrenaline: µg/kg/min, dopamine: µg/kg/min.

The APACHE II and the SOFA scores prior to ECMO initiation were 21 ± 2.64 and 11.33 ± 2.88, respectively, in decannulated patients. The same scores were 24.6 ± 4.98, and 8.20 ± 3.7, respectively, in nondecannulated patients. In the 24th hour of ECMO, the APACHE II and SOFA scores were 19.67 ± 4.5 and 13 ± 4.35, respectively, in decannulated patients, and 31.2 ± 10.33 and 11.6 ± 2.60, in nondecannulated patients. 

## 4. Discussion

To our knowledge, our center is the first and only Emergency Department (ED) to perform ECMO in Turkey, including all stages of ECMO: patient selection, cannulation, running, weaning, and discharge. This paper reports our experience with ECMO in the CCU of our ED. As a reflection of the variety of patients who are admitted to our ED, our study population consists of patients with various presentations and etiologies, such as intoxications (Table 1). 

In a study of intoxicated patients [8], the authors found that the outcome of the survival rate was significantly worse in patients with persistent acidosis (in the 24th-hour blood gas analysis). Because of the relatively small sample size and the heterogeneity of our study population, evaluating whether these parameters are associated with poor prognosis or not might be misleading. However, we observed an increase in the lactate levels after the first hour in deceased patients in our study (Table 2). The pathophysiology of lactate is much debated, although it is currently acknowledged that lactate represents more than merely the anaerobic processes [9]. The only exception to the increase of the lactate level after the first hour was seen in Case 8 of our study. However, the lactate began to rise on the 7th day, and the patient swiftly deteriorated and died within the following day. Overall, our observation of the increase in lactate was consistent with the general medical knowledge and may be valuable for the prognosis of ECMO patients.

We observed a decrease in inotropic and vasopressor drug infusion demand after the ECMO initiation (Table 3). In addition to the low demand of vasopressors in Case 4 compared to other patients, this decrease was significant especially in the patients who survived decannulation (Cases 5, 6, and 7). However, complications had a major influence on mortality among these patients. Case 4 developed severe fungemia, and Case 7 developed HIT after the decannulation, leading both patients to death. Case 5 had in-hospital cardiac arrest without a history of comorbidities, and Case 6 did not have any cardiac compromise that leads to a need for vasopressors.

The APACHE II scores in patients who survived decannulation prior to ECMO were relatively low compared to the patients who could not be decannulated. Furthermore, the APACHE II scores in the 24th hour of ECMO decreased in this group. By contrast, the SOFA scores were on the rise during this period. Various scoring systems have been investigated in critically ill patients. In a Chinese single-center study [10], the APACHE II score was found to have a broader application than the SOFA score did when selecting ARDS patients who may benefit from VV ECMO. In a study [11] that compared the prognostic markers in a medical ICU, the APACHE II scoring system yielded better discrimination power than the SOFA system did. In light of these data, we suggest that the APACHE II score may be used to predict the probability of survival after decannulation in patients who are receiving ECMO therapy. 

Recent reports Extracorporeal Life Support Organization (2019). International Summary [online]. Website https://www.elso.org/Registry/Statistics/InternationalSummary.aspx [accessed 7 October 2019]. have shown that the survival to discharge or transfer rate was 29% in E-CPR, 59% in patients who received pulmonary support, and 43% in patients who received cardiac support. In our study, Cases 5, 6, and 7 survived decannulation, and Cases 5 and 6 were discharged. We think that several factors may have contributed to the low survival rate of 25%. One factor is that the ECMO device was not readily available at our institution, and when a patient was considered a candidate for extracorporeal support, the delay that was required to obtain the device was inevitable. This setback particularly had a role in Case 3, a patient who received E-CPR. A recently published metaanalysis [12] suggested that, compared to conventional CPR, E-CPR showed better results in survival and neurological outcomes, especially 3 to 6 months after the arrest. However, we believe that the lack of availability of the ECMO device running on stand-by in the resuscitation bay, especially in Turkey, is a major obstacle to accomplishing such results. In a study [13], it was reported that the duration of the mean low-flow state, particularly over 50 min, is an independent predictor of mortality. In Case 3, which was our first and the only E-CPR case, the duration of the low-flow state was around 90 min. 

ECMO provides time for the treatment of underlying reversible causes and thus acts as a bridge to therapy. However, survival depends on the timing of the reversal of these causes as well as the timing of the initiation of ECMO [14,15]. Therefore, survival will be better in cases in which underlying causes are quickly determined and treated. In our study, Cases 4 and 8 (aged 30 and 42, respectively) were initially diagnosed with ARDS due to potential H1N1 infection. Nevertheless, empirical antibiotics were promptly initiated because a nosocomial infection could not be excluded. The initial blood and sputum cultures and screening for viral pathogens were all negative. However, the pathogen could only be detected on the 5th to 6th days of their CCU stays ( *Aspergillus fumigatus* in Case 4 and a resistant strain of *Pseudomonas* in Case 8). Thus, a pathogen-specific drug administration was delayed unintentionally, which might have significantly impacted their survival. The patients were not able to survive because they lacked adequate and timely treatment.

In a case series [16] of eight E-CPR patients, where ECMO was initiated in the ED according to an algorithm, a survival rate of 27% was reported. Considering that emergency physicians provide the first medical contact in all critical patients, including those with cardiac arrest, prompt patient selection and ECMO initiation by emergency physicians might improve survival rates. This suggestion complies with a recently published review [17] where the authors stated that ECMO may be a useful tool in the hands of emergency physicians when a life-threatening situation is present. Furthermore, well-established ED ECMO programs for OHCA patients exist in the literature [18]. In this context, ECMO is gradually becoming an integral part of the ED, and we suggest that CCUs be positioned in EDs with ECMO capabilities and train staff, the combination of which will contribute to the survival of critically ill patients who need ECMO. Such a unit could also decrease the risks associated with transport to the ICU or another hospital with an available ICU bed. Moreover, by having these CCUs functioning, ED ECMO algorithms may be evaluated and tailored not only for OHCA patients, but also for all patients who may be potential ECMO candidates because of the possibility of more prompt patient selection.

The major limitation of this study is the small sample size, making the implications of this study based on mostly observational data. Only partial statistics could be done, and the only statistically significant effect of ECMO in the early phase was the decrease in carbon dioxide levels though this decrease was foreseeable due to the general physiology of the ECMO. We may assume that a larger sample size would better verify or refute our findings statistically.

## 5. Conclusions

An improvement in vital signs, laboratory markers, and a decrease in vasopressor drug demand can be achieved in the early phase of ECMO. Emergency physicians who make the first contact with critical patients should be more familiar with ECMO as resuscitation tends to be more ‘extracorporeal’ in the future. The concept of ED ECMO may be extended for selected patients who also need resuscitation with an indication other than a cardiac arrest as the early initiation of ECMO is associated with better survival rates.

## Informed consent

The study protocol received institutional review board approval (Approval no. 2019/109), and all the participants gave written informed consent in the format required by the relevant authorities.
